# Supplementary material for: Simulation-driven design of stabilized SARS-CoV-2 spike S2 immunogens
Source: Nat Commun. 2024 Aug 27;15:7370. doi: 10.1038/s41467-024-50976-9 (PMC11350062; doi:10.1038/s41467-024-50976-9)
Supplement: Supplementary file 3 — Description of Additional Supplementary Files [file 41467_2024_50976_MOESM3_ESM.pdf]

Description of Additional Supplementary Files

File Name: Supplementary Movie 1

Description: Opening of HexaPro-SS-Δstalk S2 trimer. The movie shows the closed-to-open transition of HexaPro-SS-Δstalk as obtained from the respective WE MD simulation. Top-down (left) and side (right) views are provided. The S2 trimer is illustrated with a surface representation, where the three protomers are colored with varying shades of purples. The CHs and HR1 helices are depicted as cartoons. Glycans are shown with gray sticks. The opening pathway shown in the movie corresponds to the pathway highlighted in Figure 2.

File Name: Supplementary Movie 2

Description: Stabilization of the S2 trimer's closed prefusion conformation in HexaPro-SS-2W. The movie highlights the stabilizing role played by V991W and T998W introduced in HexaPro-SS-2W. An unsuccessful pathway, i.e., a WE MD trajectory where only closed conformations were sampled, extracted from the respective WE simulation, is also shown. The S2 trimer is illustrated with cartoons colored in varying tones of purple according to the respective chain (A being the lightest, C being the darkest). Side chains of residues contributing to forming hydrophobic packing and D994–R995 salt bridge at the S2 trimer apex are illustrated as sticks. Glycans are shown with gray sticks. Solid lines connect residues that form hydrophobic (π–π) interactions, whereas dashed lines indicate electrostatic (cation–π and salt bridge) interactions.

File Name: Supplementary Data 1

Description: S2 trimer's WE opening trajectories (.dcd, one per system) and traces (.txt, all successful pathways), WE configuration files (.cfg), summary of WE simulations (.txt), and representative closed prefusion conformations of HexaPro-SS-2W obtained from the WE simulation (.dcd).
