# Supplementary figures and images for: Simulation-driven design of stabilized SARS-CoV-2 spike S2 immunogens
Source: Nat Commun. 2024 Aug 27;15:7370. doi: 10.1038/s41467-024-50976-9 (PMC11350062; doi:10.1038/s41467-024-50976-9)

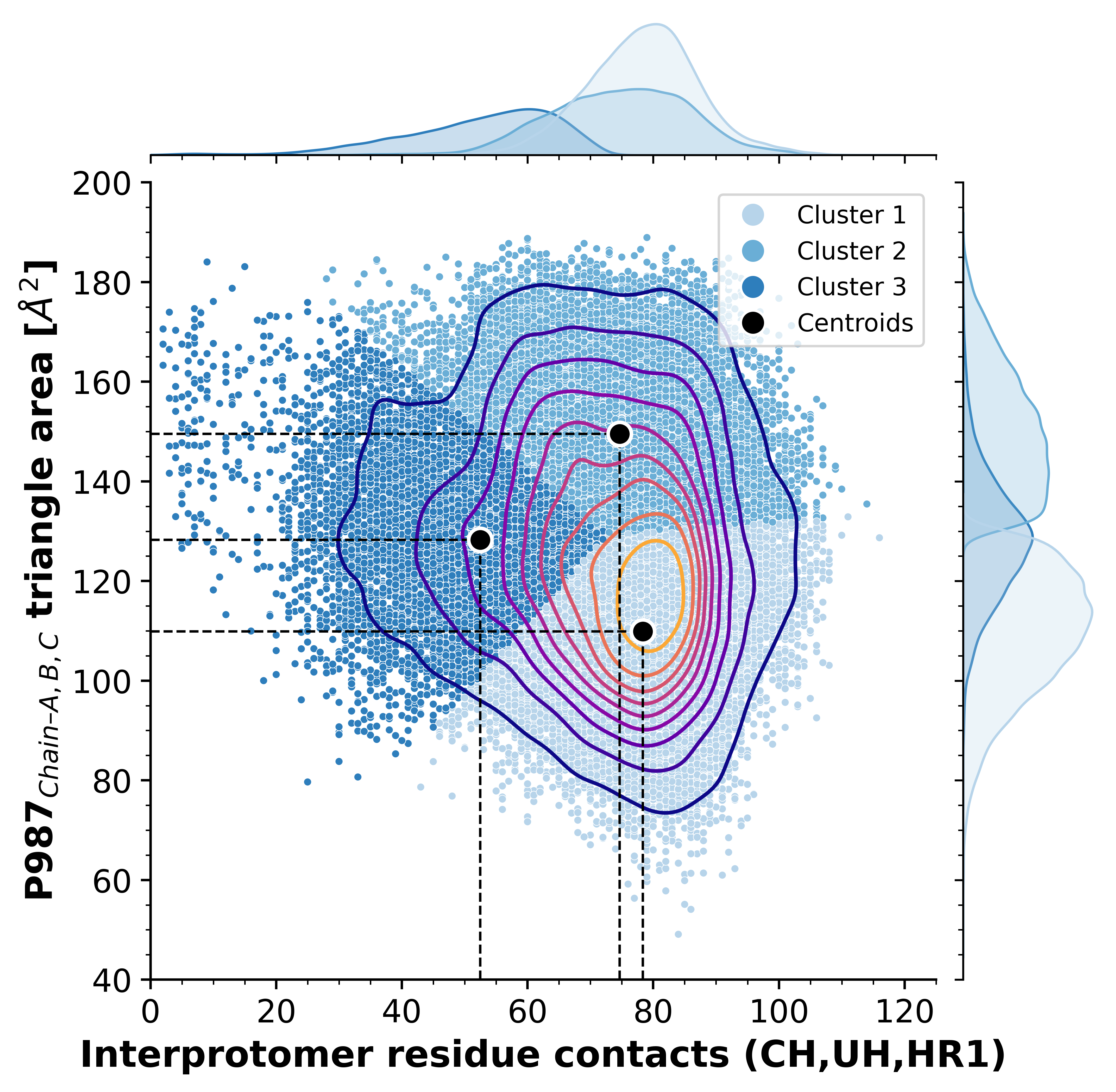

Supplement: Supplementary file 6 — Supplementary Data 1 [file 41467_2024_50976_MOESM6_ESM.zip › HexaPro-SS-2W_closed_conformations/HexaPro-SS-2W_clustering_WE_closed_conformations.png]

## Slide 1
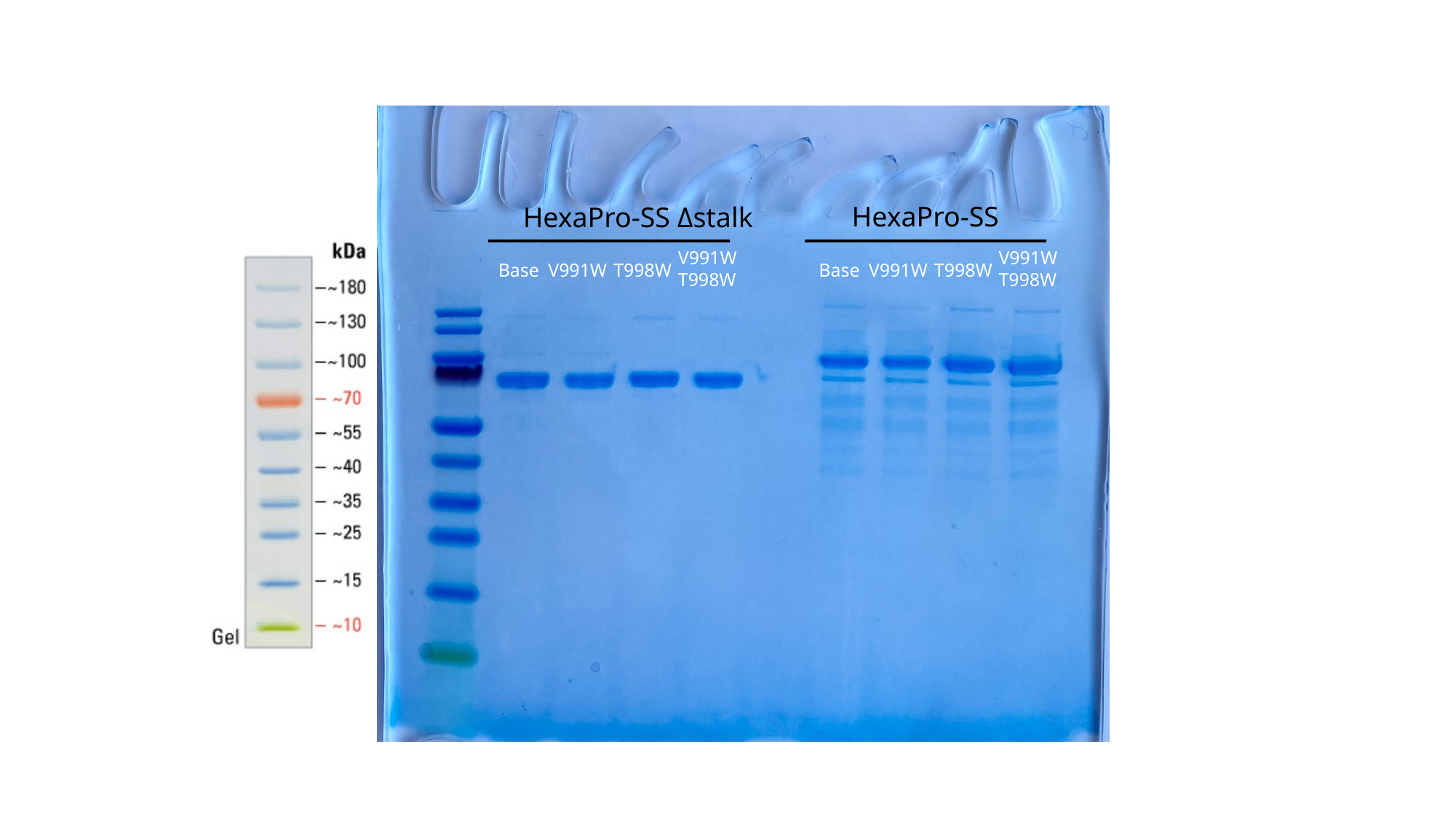

HexaPro-SS
HexaPro-SS Δstalk
V991W
T998W
V991W
T998W
Base
V991W
T998W
Base
V991W
T998W

Supplement: Supplementary file 8 — Source Data [file 41467_2024_50976_MOESM8_ESM.zip › Source_Data/figure_5a.pptx]
