# Supplementary material for: Simulation-driven design of stabilized SARS-CoV-2 spike S2 immunogens
Source: Nat Commun. 2024 Aug 27;15:7370. doi: 10.1038/s41467-024-50976-9 (PMC11350062; doi:10.1038/s41467-024-50976-9)
Supplement: Supplementary file 7 — Reporting Summary [file 41467_2024_50976_MOESM7_ESM.pdf]

nature portfolio

Corresponding author(s): Rommie Amaro

Last updated by author(s): 3/5/2014

Reporting Summary

Nature Portfolio wishes to improve the reproducibility of the work that we publish. This form provides structure for consistency and transparency in reporting. For further information on Nature Portfolio policies, see our Editorial Policies and the Editorial Policy Checklist.

Statistics

For all statistical analyses, confirm that the following items are present in the figure legend, table legend, main text, or Methods section.

n/a	Confirmed	
☐	☒	The exact sample size (*n*) for each experimental group/condition, given as a discrete number and unit of measurement
☐	☒	A statement on whether measurements were taken from distinct samples or whether the same sample was measured repeatedly
☐	☒	The statistical test(s) used AND whether they are one- or two-sided<br>*Only common tests should be described solely by name; describe more complex techniques in the Methods section.*
☐	☒	A description of all covariates tested
☐	☒	A description of any assumptions or corrections, such as tests of normality and adjustment for multiple comparisons
☐	☒	A full description of the statistical parameters including central tendency (e.g. means) or other basic estimates (e.g. regression coefficient) AND variation (e.g. standard deviation) or associated estimates of uncertainty (e.g. confidence intervals)
☐	☒	For null hypothesis testing, the test statistic (e.g. *F*, *t*, *r*) with confidence intervals, effect sizes, degrees of freedom and *P* value noted<br>*Give P values as exact values whenever suitable.*
☒	☐	For Bayesian analysis, information on the choice of priors and Markov chain Monte Carlo settings
☒	☐	For hierarchical and complex designs, identification of the appropriate level for tests and full reporting of outcomes
☒	☐	Estimates of effect sizes (e.g. Cohen's *d*, Pearson's *r*), indicating how they were calculated

Our web collection on statistics for biologists contains articles on many of the points above.

Software and code

Policy information about availability of computer code

Data collection	NAMD 3, NAMD 2.14, GROMACS 2022.1, AMBER 20, Serial EM 3.9.0 beta, Gen5 Version 3.12.08 (Agilent Technologies).
Data analysis	WESTPA 2.0, PMX, VMD 1.9.4, ChimeraX 1.6.1, PyContact, MDAnalaysis, CryoSPARC v4.0.2, DeepEMhancer, Cosmic2, Matplotlib, PHENIX 1.20.1-4487, Coot 0.9.8.3, GraphPad Prism Version 9.3.1 , GraphPad Prism Version 9.5.1 (528), cryoSPARC v4.0.2, Isolde 1.6.0, Data Analysis 11.1

For manuscripts utilizing custom algorithms or software that are central to the research but not yet described in published literature, software must be made available to editors and reviewers. We strongly encourage code deposition in a community repository (e.g. GitHub). See the Nature Portfolio guidelines for submitting code & software for further information.

Data

Policy information about availability of data

All manuscripts must include a data availability statement. This statement should provide the following information, where applicable:

- Accession codes, unique identifiers, or web links for publicly available datasets
- A description of any restrictions on data availability
- For clinical datasets or third party data, please ensure that the statement adheres to our policy

Trajectories of S2 trimer opening pathways, WE configuration files, and representative closed prefusion conformations of HexaPro-SS-2W are included in the article as Supplementary Data 1. Full datasets (all simulation input files and trajectories) for conventional MD, GaMD, WE MD, and free energy calculations are available for

download on the Amaro Lab website https://amarolab.ucsd.edu/covid19.php. Source data are provided with this paper. The atomic coordinates of HexaPro-SS-2W are available in the Protein Data Bank under PDB ID: 8VAO. The cryo-EM map is available from the Electron Microscopy Data Bank under accession code EMDB-43097.

Research involving human participants, their data, or biological material

Policy information about studies with [human participants or human data](). See also policy information about [sex, gender (identity/presentation), and sexual orientation]() and [race, ethnicity and racism]().

Reporting on sex and gender	*Use the terms sex (biological attribute) and gender (shaped by social and cultural circumstances) carefully in order to avoid confusing both terms. Indicate if findings apply to only one sex or gender; describe whether sex and gender were considered in study design; whether sex and/or gender was determined based on self-reporting or assigned and methods used. Provide in the source data disaggregated sex and gender data, where this information has been collected, and if consent has been obtained for sharing of individual-level data; provide overall numbers in this Reporting Summary. Please state if this information has not been collected. Report sex- and gender-based analyses where performed, justify reasons for lack of sex- and gender-based analysis.*
Reporting on race, ethnicity, or other socially relevant groupings	*Please specify the socially constructed or socially relevant categorization variable(s) used in your manuscript and explain why they were used. Please note that such variables should not be used as proxies for other socially constructed/relevant variables (for example, race or ethnicity should not be used as a proxy for socioeconomic status). Provide clear definitions of the relevant terms used, how they were provided (by the participants/respondents, the researchers, or third parties), and the method(s) used to classify people into the different categories (e.g. self-report, census or administrative data, social media data, etc.) Please provide details about how you controlled for confounding variables in your analyses.*
Population characteristics	*Describe the covariate-relevant population characteristics of the human research participants (e.g. age, genotypic information, past and current diagnosis and treatment categories). If you filled out the behavioural & social sciences study design questions and have nothing to add here, write "See above."*
Recruitment	*Describe how participants were recruited. Outline any potential self-selection bias or other biases that may be present and how these are likely to impact results.*
Ethics oversight	*Identify the organization(s) that approved the study protocol.*

Note that full information on the approval of the study protocol must also be provided in the manuscript.

Field-specific reporting

Please select the one below that is the best fit for your research. If you are not sure, read the appropriate sections before making your selection.

☒ Life sciences ☐ Behavioural & social sciences ☐ Ecological, evolutionary & environmental sciences

For a reference copy of the document with all sections, see [nature.com/documents/nr-reporting-summary-flat.pdf]()

Life sciences study design

All studies must disclose on these points even when the disclosure is negative.

Sample size	For each group, n=6 was used. A post-hoc power analysis shows that at an alpha = 0.05, an ANOVA test has an effect size of 0.95 (nearly 1 STD) with a power of 0.91.
Data exclusions	Select %infectivity values were excluded from neutralization data analysis when they did not correspond with an apparent trend determined by other replicates in the group or by values at dilution points lower or higher than the one in question. One mouse was excluded from AUC of rVSV-SARS-CoV2 (Omicron) neutralization analysis due to the lack of sera; the animal was euthanized during blood draws for humane reasons before enough blood could be collected.
Replication	Having multiple animals per group served as our replication. Each neutralization experiment was conducted once with each virus.
Randomization	C57BL/6J mice were randomly allocated to immunization groups.
Blinding	Investigators were not blind to group allocation of mice.

Reporting for specific materials, systems and methods

We require information from authors about some types of materials, experimental systems and methods used in many studies. Here, indicate whether each material, system or method listed is relevant to your study. If you are not sure if a list item applies to your research, read the appropriate section before selecting a response.

Materials & experimental systems

n/a	Involved in the study	
☐	☒	Antibodies
☐	☒	Eukaryotic cell lines
☒	☐	Palaeontology and archaeology
☐	☒	Animals and other organisms
☒	☐	Clinical data
☒	☐	Dual use research of concern
☒	☐	Plants

Methods

n/a	Involved in the study	
☒	☐	ChIP-seq
☒	☐	Flow cytometry
☒	☐	MRI-based neuroimaging

Antibodies

Antibodies used	Antibody sequences were obtained from published manuscripts and were expressed and purified internally
Validation	IgG22: Hsieh, C. L. et al. Stabilized coronavirus spike stem elicits a broadly protective antibody. Cell Rep. 37, 109929 (2021). S2P6: Pinto, D. et al. Broad betacoronavirus neutralization by a stem helix–specific human antibody. Science (80-.). 373, 1109–1116 (2021). CoV44-79 and CoV91-27: Dacon, C. et al. Broadly neutralizing antibodies target the coronavirus fusion peptide. Science (80-.). 377, 728–735 (2022). CC40.8: Silva, R. P. et al. Identification of a conserved S2 epitope present on spike proteins from all highly pathogenic coronaviruses. Elife 12, (2023). N3-1: Goike, J. et al. SARS-COV-2 Omicron variants conformationally escape a rare quaternary antibody binding mode. Communications Biology 6, 1250 (2023).

Eukaryotic cell lines

Policy information about cell lines and Sex and Gender in Research

Cell line source(s)	FreeStyle-293 cells (Invitrogen), Vero cells (ATCC), 293FT (Thermo Fisher Scientific)
Authentication	Cells were purchased commercially and were not further validated.
Mycoplasma contamination	Confirmed that all cell lines are free of mycoplasma by PCR
Commonly misidentified lines (See ICLAC register)	N/A

Animals and other research organisms

Policy information about studies involving animals; ARRIVE guidelines recommended for reporting animal research, and Sex and Gender in Research

Laboratory animals	6-8 week old female C57BL/6J mice from the Jackson Laboratory
Wild animals	N/A
Reporting on sex	Sex was considered in the study design. A mouse-adapted SARS-CoV-2 strain (MA-10) (Dinnon, III et al., 2020) was previously developed in female mice. To remain consistent for our immunization studies, we used female mice. Sex-based analyses were not performed.
Field-collected samples	N/A
Ethics oversight	The study protocol was approved by the Albert Einstein College of Medicine Institutional Animal Care and Use Committee.

Note that full information on the approval of the study protocol must also be provided in the manuscript.

Plants

Seed stocks

Report on the source of all seed stocks or other plant material used. If applicable, state the seed stock centre and catalogue number. If plant specimens were collected from the field, describe the collection location, date and sampling procedures.

Novel plant genotypes

Describe the methods by which all novel plant genotypes were produced. This includes those generated by transgenic approaches, gene editing, chemical/radiation-based mutagenesis and hybridization. For transgenic lines, describe the transformation method, the number of independent lines analyzed and the generation upon which experiments were performed. For gene-edited lines, describe the editor used, the endogenous sequence targeted for editing, the targeting guide RNA sequence (if applicable) and how the editor was applied.

Authentication

Describe any authentication procedures for each seed stock used or novel genotype generated. Describe any experiments used to assess the effect of a mutation and, where applicable, how potential secondary effects (e.g. second site T-DNA insertions, mosiacism, off-target gene editing) were examined.
